# Current Status of Partial Laryngeal Surgery for Advanced Laryngeal Cancer: When and Why?

**DOI:** 10.1007/s11912-024-01516-7

**Published:** 2024-04-22

**Authors:** Erika Crosetti, Marco Fantini, Ilaria Bertotto, Andy Bertolin, Giulia Arrigoni, Andrea Lorenzi, Giovanni Succo

**Affiliations:** 1 ENT Clinic – Head and Neck Cancer Unit, San Giovanni Bosco Hospital, Turin, Italy; 2 Department of Oncology, University of Turin, Turin, Italy; 3 Radiology Department, Candiolo Cancer Institute – IRCCS, Candiolo, TO Italy; 4 ENT Department, Vittorio Veneto Hospital, AULSS 2 Marca Trevigiana, Treviso, Italy

**Keywords:** Laryngeal cancer, Partial laryngectomy, OPHL, Partial laryngeal surgery

## Abstract

**Purpose of Review:**

This paper aims to evaluate the evolution and current status of partial laryngeal surgery in the treatment of advanced laryngeal cancer (LC). Specifically, recent progress in the selection of both patients and tumors, together with surgical and rehabilitation innovations, have contributed to balancing oncological control with the maintenance of quality of life in naïve and radiorecurrent patients. The main aspect is represented by the recognized role of open partial horizontal laryngectomies (OPHLs) in this new era of laryngeal cancer treatment.

**Recent Findings:**

Recent advancements highlight OPHLs’ efficacy for conservative management of intermediate to advanced stages of LC. Innovations such as supratracheal partial laryngectomy have expanded surgical options, offering a modular approach to complex cases. Improved understanding of tumor biology, enhanced imaging techniques, and more precise preoperative planning have led to better patient outcomes, emphasizing the importance of a conservative function-preserving surgical treatment. These advancements reflect a broader trend towards individualized treatment plans that prioritize both survival and quality of life.

**Summary:**

OPHLs play an important role in current management of intermediate/advanced LC, effectively balancing oncological control with the preservation of laryngeal functions. Critical factors include meticulous patient and tumor selection, the impact of surgical and technological refinements on functional outcomes, and the necessity of a multidisciplinary approach in treatment planning. Current evidence justifies the use of these interventions in many intermediate T-stage laryngeal tumors, even at risk of upstaging on pathological examination. The oncological results, the preservation of laryngeal function and the laryngectomy-free survival achieved with OPHLs appear to be highly competitive with those of non surgical organ-preservation protocols, aiming to introduce a new standard in the LC treatment.

## Introduction

The surgical landscape for advanced laryngeal cancer (LC) has undergone significant transformations since the first total laryngectomy, carried out in 1873, marking over 150 years of evolution toward strategies aiming to the preservation of the larynx and its functions [1]. Initially, the focus in the treatment of LC was predominantly on achieving loco-regional control, with the preservation of the larynx’s function being of secondary importance. However, as knowledge of the natural history and epidemiology of laryngeal cancer deepened, the paradigm shifted. The latter half of the twentieth century witnessed the zenith of the popularity of partial laryngeal surgery, especially for early- and some well selected intermediate-stage tumors. These less invasive procedures enabled the maintenance of the larynx’s critical functions, including voice, airway protection, and swallowing [2–5].

This era of surgical optimism gradually diminished as the century drew to a close, with the advent of organ preservation protocols involving chemoradiation [6, 7]. These protocols held the promise not only of enhancing locoregional control of the disease but also of improving the quality of life for patients through the preservation of the larynx, even in more advanced stages. Despite the ready enthusiasm, however, long-term follow-up studies disclosed limitations in terms of overall survival and laryngectomy-free survival rates, necessitating a reevaluation of the surgical approaches to LC [8]. A resurgence of interest in partial laryngeal surgery has followed, driven by a more refined understanding of tumor biology, precise patient selection criteria, advances in surgical techniques and a modular approach to surgery, adapted to the extent of the tumor [9, 10]. This emerging paradigm elevates the priorities to encompass not merely survival but also the preservation of laryngeal function, thus responding adeptly to the challenges presented by intermediate to advanced-stage primary tumors and the failures after radiotherapy and laser surgery [11].

Within this context, open partial horizontal laryngectomies (OPHLs) have emerged as the workhorse of contemporary surgical efforts to preserve laryngeal function. These procedures, which have evolved over the past few decades, have proven their efficacy in treating intermediate and locally advanced LC without significant neck metastases [12•]. This review aims to explore the status of partial laryngeal surgery, specifically focusing on its application for advanced LC. It will examine the strategic shift from total laryngectomy toward modular function-preserving surgical interventions, emphasizing the renewed focus on achieving a balance between oncological control and good quality of life.

## Clinical and Pathological Features Favoring Surgical Laryngeal Function Preservation

Modern partial laryngeal surgery has evolved because it has learned to better exploit the specific clinicopathological characteristics of tumors, thus expanding the indications for surgery to include intermediate/advanced T-stage tumors. This adaptation allows for more patients with advanced disease to benefit from surgeries that aim to preserve laryngeal function. The key factors are:

1. Low incidence of cervical lymph nodes metastases in intermediate/advanced glottic cancer.

Advanced glottic LCs are often characterized by a reduced likelihood of developing metastases to cervical lymph nodes. This characteristic strongly supports the use of surgical strategies focused on preserving laryngeal function. It enables the treatment of LC by surgical approach alone, even in advanced stages, thereby significantly improving the quality of life by preserving voice and swallowing functions [13].

2. Understanding local spreading patterns of T3 and T4a LC.

The local spread of advanced LC exhibits specific patterns. T3 tumors are associated with the involvement of the laryngeal visceral spaces, suggesting a deeper invasion within the laryngeal structure, while T4a tumors show expansion beyond the larynx, characterized by varying degrees of transcartilaginous or transmembranous progression into extralaryngeal tissues. The preoperative identification of these patterns is essential for planning very precise surgical interventions aimed at selectively removing the laryngeal sites involved by cancer, preserving the anatomical structures essential for maintaining function [14, 15].

3. Pre-operative stratification of advanced tumors based on topographical criteria.

The concept of pre-operative stratification of advanced tumors based on topographical criteria, particularly the “magic plane” concept, was a significant advancement introduced by researchers aiming to refine the oncological and functional outcomes of partial surgery on advanced LC. This method, which focuses on the tumor’s spreading into the posterior paraglottic space and considers the functional criterion of arytenoid mobility, has been pivotal in enhancing the precision of surgical indications, resulting in their broader extension in anterior, posterior, and inferior directions. By integrating both anatomical and functional assessments, surgery can more accurately tailored, significantly improving the outcomes of laryngeal preservation surgeries [12•, 16••, 17•].

4. Distinguishing between different patterns of arytenoid fixity.

Although traditionally this functional aspect has been considered a highly negative prognostic factor for partial surgery, the analysis of different patterns of spreading in relation to arytenoid cartilage fixity has revealed that some of these patterns may still be compatible with laryngeal preservation surgery with a good prognosis. The ability to differentiate these patterns allows for more precise and safe patient selection [18•].

5. Patient selection based on clinical and demographic parameters.

The selection process for candidates undergoing partial laryngeal surgery involves more than just evaluating the tumor’s physical traits; it incorporates a comprehensive review of clinical and demographic factors. This strategy facilitates the identification of patients suitable for a challenging surgery and achieve an effective recovery while preserving laryngeal functions. Considerations include the patient’s general health, age and ability to recover from possible postoperative complications, aiming for successful recovery and maintenance of functions [19•].

## Advances in Clinic and Radiologic Work-up

The advancements in the management of locally advanced LC underscore the critical role of a meticulous endoscopic and radiological work-up. Recent significant improvements in the endoscopic work-up offer a suite of sophisticated techniques, such as HD videolaryngoscopy, narrow-band imaging (NBI), and SPIES (STORZ professional image enhancement system). These diagnostic tools allow detailed visualization of the mucosal surface and the vascular architecture, facilitating early detection of neoplastic lesions and accurate assessment of their superficial extent, particularly at the lesion’s boundaries [20, 21].

In the optimal pathway for planning an OPHL, office-based endoscopy plays a critical role. It enables the evaluation of the tumor’s superficial extension, highlighting potential pathways of spread as well as the mobility of the vocal cords and arytenoids [18•, 22].

Imaging, including computed tomography (CT) and magnetic resonance imaging (MRI), is indispensable for understanding the patterns of submucosal spread of the disease. The CT scan, because of its rapid image acquisition, high spatial resolution, and widespread availability, is considered the cornerstone of LC staging. It is especially beneficial for assessing cartilage involvement and detecting extralaryngeal tumor spreading, crucial elements in surgical planning [23••]. Conversely, MRI provides superior soft-tissue contrast, enabling an in-depth analysis of submucosal tumor spread, cartilage irregularities, and the involvement of visceral spaces, such as the paraglottic and pre-epiglottic spaces [24••, 25]. Such information is of paramount importance for accurately defining tumor extension and deciding on conservative surgical approaches, such as OPHL [26•]. Adding diffusion-weighted imaging (DWI) to MRI enhances its diagnostic power by differentiating between peritumoral edema and tumor, which is particularly important for a more accurate definition when considering an OPHL as a salvage treatment for radiorecurrent tumors [27•].

**Fig. 1:**
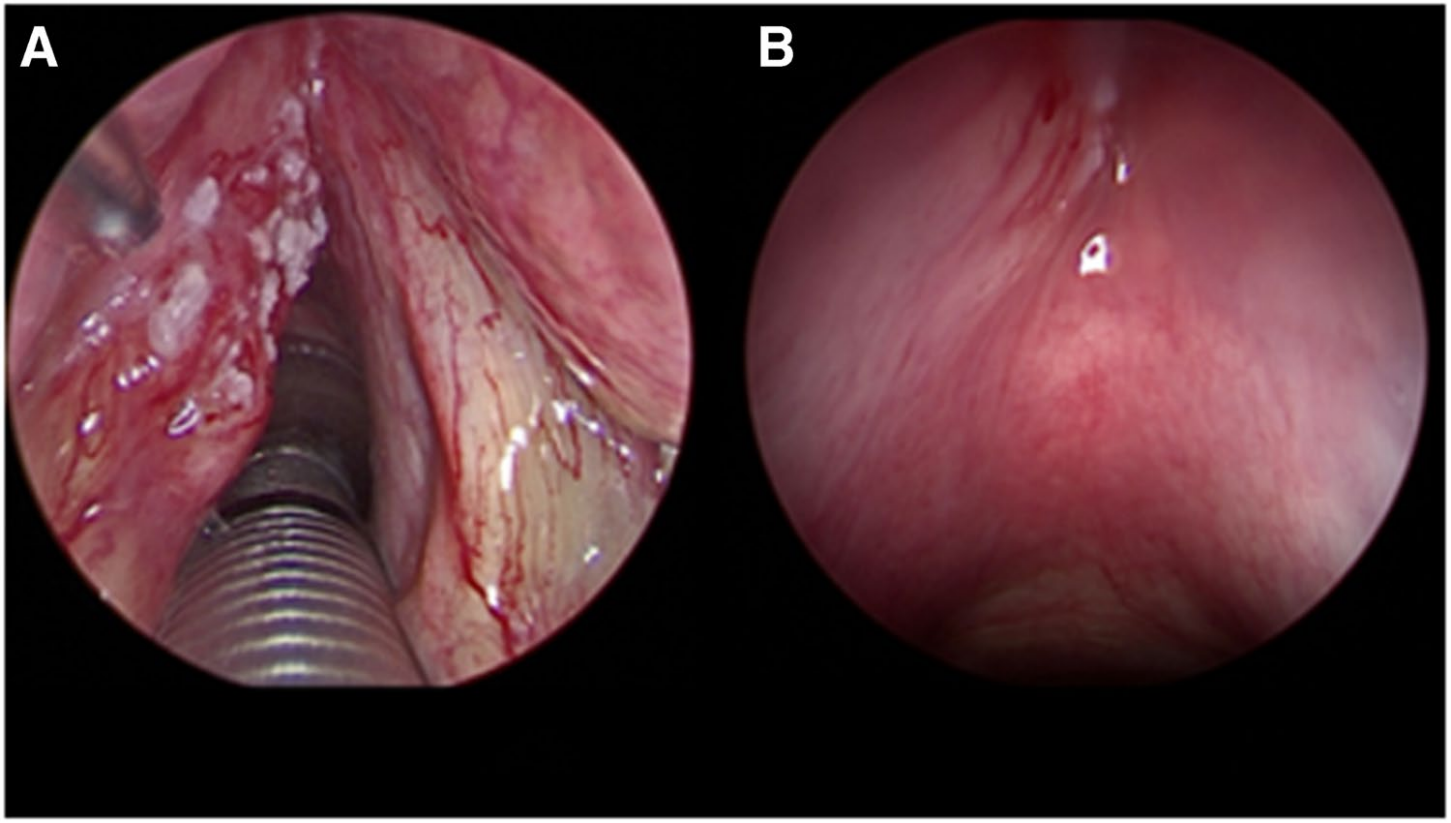
Endoscopic work-up in narcosis: **A** glottic cancer involving the left vocal cord and determining impairment of the vocal cord motility (O^ 5 mm telescope). **B** Same patient: a slight submucosal swelling with evident tributary vessel in the left anterior subglottic area is noticeable. Tumor highly suspected for spreading through the cricothyroid membrane (70° 5 mm telescope)

Endoscopy under general anesthesia completes the work-up by providing a final analysis of the tumor’s growth patterns in less visible sites, allowing for lesion palpation and targeted biopsies. This step is crucial for understanding the three-dimensional aspects of the lesion and anticipating the type of surgery within a modular surgical approach (Fig. 1).

**Fig. 2:**
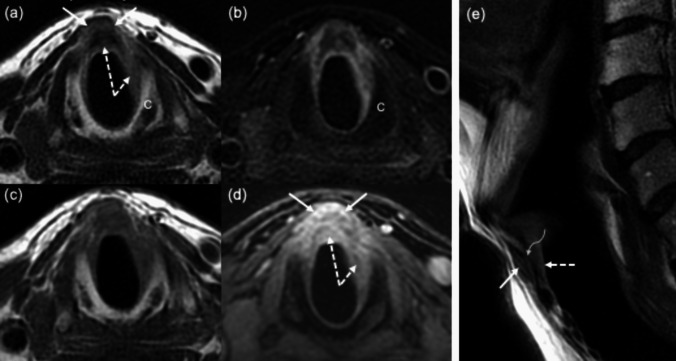
Subglottic level on axial FSE T2-weighted (**a**), FSE fat sat T2-weighted (**b**), FSE T1-weighted (**c**) and 3D gradient echo fat sat T1 post contrast (**d**); FSE T2-weighted on sagittal plane (**e**). A neoplastic submucosal thickening (white dashed arrows) with enhancement (**d**) in the anterior and left side of subglottic level. At the same level similar soft tissue representing extralaryngeal spread (white arrows) through the cricothyroid membrane (**e**) (curved arrow) is present. The cricoid cartilage (C) does not show abnormal alterations indicative of invasion. FSE, fast spin echo

A strict collaboration between clinicians and radiologists in the diagnostic work-up is mandatory, particularly when facing advanced and more challenging cases (Fig. 2). In our experience, the growth of expertise resulting from a correct selection must emerge from a continuous cyclical information exchange between clinician-radiologist and pathologist, based on precise questions and consequent answers, as recently well described by Crosetti et al. [28].

## Recent Surgical Advances

Recent literature demonstrates that systematic approaches to treating locally advanced tumors have necessitated a refinement of the classic principles of partial laryngeal surgery. This evolution highlights the need for modular surgical strategies that ensure the radical removal of tumors, even in the face of complex cases [29].

The surgical toolkit for advanced LC has recently been significatively improved by the addition of supratracheal partial laryngectomy (STPL), marking a significant advancement in treating glottic tumors with anterior, inferior and/or posterior subglottic extensions, even when there is a risk of extralaryngeal spread [30]. This innovation broadens the scope of surgical options, offering a comprehensive approach to even the most complex cases [31••].

Traditionally, supraglottic and supracricoid laryngectomies were the mainstays for highly selected, locally advanced tumors of the supraglottic and glottic sites, including those with transglottic spread. The goal of introducing STPL is to enhance the surgical intervention’s radicality in treating challenging tumor locations, enhancing the potential for tumor removal while striving to preserve as much laryngeal function as possible [32••].

The introduction of STPL and its demonstrated oncological and functional success have paved the way for the new European Laryngological Society (ELS) classification of OPHL, as proposed by Succo et al. in 2014 [33]. This classification organizes OPHLs into three categories based on the surgical resection’s lower boundary, each designed to preserve laryngeal function. This classification aimed to facilitate a modular, conservative surgical approach to laryngeal cancer, providing surgeons with the flexibility to choose within twelve distinct partial horizontal open neck procedures. This innovative framework represents a significant step forward in the surgical management of laryngeal cancer, promoting both oncological efficacy and the preservation of patients’ quality of life [29].

The minimal resection margins achieved, in certain patterns of spreading, with these surgeries, if found to be disease-free as a result of a meticulous and standardized examination of the surgical specimen, are sufficient to achieve favorable oncological outcomes, even in locally advanced tumors [34].

In managing locally advanced tumors, it is also crucial to recognize the increased risk of lymphatic metastasis to level VI, especially in tumors with subglottic spread and anterior extralaryngeal extension, making level VI dissection critically important [35••]. Additionally, given the risk of unrecognized extralaryngeal extension in advanced tumors, strap muscle resection becomes vital to achieve anteriorly a level of radicality comparable to total laryngectomy. This surgical strategy, as demonstrated by Schindler’s extensive research, ultimately does not compromise swallowing function [36••].

## Oncologic Results

The OPHLs have significantly improved the conservative treatment of advanced LC, delivering stable and robust outcomes. Success is especially observed in cases where tumors do not transgress the “magic plane” and do not determine arytenoid fixation, underscoring the pivotal role of anatomical and functional compartmentalization in selecting cases with a better prognosis. The predictive value of this compartmentalization surpasses traditional TNM categorization, stressing the significance of tumor location in planning treatment. Initial research highlighted that posterior T3 tumors, spreading within posterior paraglottic space and determining arytenoid fixation yield poorer outcomes when treated with OPHLs, with significantly worse oncologic outcomes compared to anterior tumors (OS *p* < 0.001, DSS *p* < 0.05, and DFS *p* < 0.001) [16••]. Subsequent studies by Del Bon et al. have reinforced these findings, demonstrating that for locally advanced T3–T4a tumors treated with OPHLs, survival outcomes significantly favor anterior over posterior tumors, marking a statistically significant difference in survival rates (OS, DSS, RFS were 91%, 94.1%, and 72.6%, respectively, for anterior tumors, and 60.3%, 66.3%, and 49.1%, respectively, for posterior lesions—statistically significant differences [17•].

Merging these insights with the range of surgical interventions unveils a clear prognostic benefit.

In another recent study, it was demonstrated that OPHL can still achieve good outcomes for tumors with posterior extension and resulting arytenoid fixation, provided that the subglottic extension measured at the midline of the vocal cord is less than 10 mm [18•].

Thanks to whole these evidences, the analysis of data from reviews on naïve T3N0 patients, showing high rates of local and locoregional control as well as remarkable 5-year overall survival rates, has become more understandable (Table 1). Campo et al. have shown that OPHLs are a viable option for patients with naïve pT3N0 LC, presenting average 5-year rates of OS, DSS, DFS, and LFS at 80.5%, 83.4%, 77.4%, and 77.9%, respectively. Moreover, OPHL’s ability to achieve high rates of laryngectomy-free and laryngoesophageal dysfunction-free survival in T3 patients (with 5-year LFS and LEDFS at 93% and 93.1% respectively for anterior T3 vs. 77.7% and 76.6% for posterior tumors) underscores its potential to equilibrate oncological effectiveness with quality-of-life considerations [37•].

**Table 1:** Studies analyzing 5 years oncologic results after OPHL for T3 and T4 LC

Authors	Year	T stage	No. of Patients	OS (%)	DSS (%)	DFS (%)	LRC (%)	LFS (%)
Laudadio et al. [40]	2006	pT3	58	88.7	NA	77.6	NA	NA
		pT4	13	61.5	NA	53.8	NA	NA
Sánchez-Cuadrado et al. [41]	2011	T3	17	52	64	67	NA	NA
Mercante et al. [42]	2013	cT3	32	87.3	NA	78.2	96.2	NA
Sperry et al. [43]	2013	T3	34	83.8	84.4	NA	85.2	92.6
Rizzotto et al. [31••]	2015	pT3	50	86.0	NA	86.0	86.0	NA
		pT4a	51	80.4	NA	60.8	62.7	NA
Succo et al. [16••]	2018	pT3	390	90.1	94.5	87.4	88.8	86.8
		pT4a	89	81.9	91.3	71.2	75.5	72.9
Xia et al. [44]	2018	T3	106	65.8	73.6	72.1	NA	NA
Zhou et al. [45]	2018	T3	106	77.1	78	64.8	NA	NA
Del Bon et al. [17•]	2019	pT3	67	74.1	80.5	63.4	NA	63.8
		pT4a	18	71.8	71.8	43	NA	43.1
Gong et al. [46]	2019	T3	42	77.8	77.8	63.3	NA	NA
Mattioli et al. [47]	2021	pT3	28	92.9	100	89.3	NA	89.3
De Vincentiis et al. [12•]	2022	T3	116	79.3	85.3	81	NA	82.76
		T4	33	70.9	77.4	77	NA	66.67
Succo et al. [38]	2023	pT4a	134	82.1	89.8	75.7	NA	93.3

*DFS* disease-free survival, *DSS* disease-specific survival, *LFS* laryngectomy-free survival, *LRC* locoregional control, *NA* not available, *OS* overall survival

Conservative management of pT4a tumors, often previously viewed as anecdotal and related to minimal extralaryngeal extension of tumors initially staged cT3 and then identified as pT4a at pathology, has benefited from recent multicenter analyses [38]. This approach for pT4a tumors with minimal extralaryngeal volume, based on type II and type III OPHLs, aligns oncological outcomes with those of lower T categories. The observation that these cases often result from understaging during the clinical work-up underscores that a systematic and structured approach to treatment can also manage more advanced and inherently riskier cases effectively (OS, DSS, DFS, FFL, LEDFS respectively 86.3%, 94.4%, 82.7%, 97%, 84.7% in clinically understaged vs 68.2%, 74.4%, 49.5%, 64.8%, 56.7% in cases showing clear extralaryngeal extension) [38]. Even in the case of pT4a tumors, these studies shed more light and allow for a better understanding of the data published in the literature by Laccoureye et al. [39], Succo et al. [11], and De Vincentiis et al. [12•].

## Functional Results

OPHL stand as a pivotal conservative surgical approach in the management of LC, extending from early-to-intermediate stages to select T4a diseases [33]. OPHLs are recognized for their oncological safety, showcasing commendable overall survival, disease-free survival, and laryngectomy-free survival rates [11, 48]. Moreover, these procedures are designed to conserve the primary functions of the larynx (swallowing, breathing, and phonation) while aiming to obviate the necessity for a permanent tracheostomy. Reports indicate 5-year laryngeal function preservation (LFP) rates post–OPHL ranging from 91.2% to 98.5% according to the primary extent of the disease, with comparable functional outcomes observed between type II and type III OPHLs [32••, 36••, 49]. Despite these promising figures, the incidence of acute complications and late sequelae, potentially necessitating total laryngectomy or permanent gastrostomy, underscores the significance of surgical expertise and patient-specific considerations.

The prognostication of functional outcomes post–OPHL is imperative for surgical planning. This entails a comprehensive evaluation of preoperative patient-related and disease-related factors to tailor individualized treatment strategies. Functional recovery, particularly concerning voice, breathing, and swallowing, generally exhibits satisfactory levels, albeit with notable variability. Voice quality preservation is predominantly associated with type I OPHLs due to the sparing of vocal folds, whereas type II and type III are linked to significant, albeit similar, deteriorations in voice quality. Nonetheless, most patients achieve acceptable levels of oral communication through substitution voice techniques [50].

Swallowing function recovery post–OPHL emerges as a formidable challenge, with immediate postoperative impairment gradually ameliorating over approximately six months, allowing most patients to return to a free oral diet. However, the persistence of chronic aspirations and postswallowing residues in some individuals heightens the risk of aspiration pneumonia. Variability in tracheal cannula removal, nasogastric tube (NGT) feeding, and hospital stay durations further exemplify the heterogeneity of functional recovery, as documented in the literature [36••, 51].

Recent analyses by Succo et al. have elucidated good LFP outcomes post–OPHL, with 5-year rates paralleling disease [11, 48]. Delineating prognostic factors for challenging functional recoveries is crucial for optimizing pre-operative patient selection. In a study by Fantini et al. factors such as advanced age, lower BMI, smoking habits, comorbidities, osteophytosis, and higher cT-staging have been significantly associated with adverse clinical and functional outcomes. Specifically, advanced cT-staging and lower BMI emerged as independent risk factors for postoperative sequelae and PEG positioning, while smoking habits and advanced age correlate with delayed NGT removal [19•].

In recent years, there has been an increasing focus not only on oncological outcomes but also on the functional results of partial laryngectomies. Beyond survival rates, there has been a growing necessity to pay close attention to the quality of life of patients undergoing OPHL. Table 2 lists some of the most significant studies from the last 5 years, specifically focusing on functional outcomes after OPHL. These studies highlight the scientific community’s keen interest in this subject, demonstrating a steadfast commitment to improving not only patient survival prospects but also their postoperative quality of life.

**Table 2:** Key studies on functional outcomes after OPHL in the past 5 years

Authors	Year	No. of patients	Summary
Gong et al. [46]	2019	164	OPHL type IIa offers reliable oncologic and functional outcomes for glottic T1b, T2, and selected T3 LC patients, with high decannulation and feeding tube removal rates
Gökmen et al. [52]	2020	50	TOLM provides better functional outcomes than OPHL in patients with supraglottic LC, showing less need for tracheotomy and shorter hospitalization
Vella et al. [53]	2020	37	Analyzes breathing, swallowing, and phonation in patients treated with OPHL type II and infrahyoid muscle reconstruction
Fantini et al. [19•]	2021	123	Examines pre-operative prognostic factors for outcomes after OPHLs, highlighting challenges in neolarynx recovery affecting functional outcome
Mesolella et al. [54]	2021	35	OPHL type II is well-tolerated with good functional outcomes for advanced LC, highlighting oncological safety and quality of life improvements
Gigot et al. [55]	2022	20	OPHL after failed RT/CT shows that it is a viable alternative to total laryngectomy, with significant speech recovery and oral diet restart
Grasso et al. [56]	2023	20	Study focuses on factors impacting post-decannulation swallowing outcomes after OPHL type II
Locatello et al. [57]	2023	NA	Review on the role of post-operative RT after OPHL in LC, finding no significant difference in overall survival with or without post-operative RT
Saturno et al. [58]	2024	193	OPHL type II shows excellent oncologic and functional outcomes, with high survival rates and low post-operative gastrostomy tube dependency

*NA* not available, *TOLM* transoral laser microsurgery

Given these assumptions, there are instances where functional outcomes may initially not meet expectations. It is crucial, however, to emphasize that unsatisfactory functional results are not definitive. A variety of rehabilitative and surgical interventions exist and can often significantly improve the primary laryngeal functions (respiratory, deglutitory, and phonatory).

Recent studies have underscored the potential for substantial functional recovery through targeted interventions. For instance, phonosurgical injection approaches have been explored as a means to restore phonation and swallowing after OPHL, demonstrating promising outcomes in voice quality and swallowing improvement [59, 60]. Similarly, the management of laryngeal stenosis post-reconstructive partial laryngectomies via transoral laser microsurgery has shown to be effective in improving airway patency and, by extension, respiratory function [61]. Additionally, the proprioceptive elastic method (PROEL) has been employed for substitution voice rehabilitation, offering a novel approach to phonatory function recovery with encouraging preliminary results [62].

These interventions illustrate the multifaceted approach to managing and enhancing functional recovery post–OPHL. The surgical techniques aim to directly address anatomical and physiological deficits, while rehabilitative strategies focus on optimizing the residual function and compensatory mechanisms. The integration of these approaches, tailored to the individual patient’s needs and the specific nature of their post-operative deficits, is key to achieving the best possible functional outcomes.

## OPHL in Radio/Laser-Recurrent Laryngeal Cancer

Research on surgical salvage options following primary treatments such as radiotherapy (RT), chemoradiation (CRT), or trans oral laser microsurgery (TOLM), has recently grown in importance. Total laryngectomy has traditionally been the go-to method for addressing failures of primary treatments. However, the efficacy of conservative surgical approaches for selected cases is now well-established. These approaches present a good alternative to total laryngectomy, offering a balance between oncological control and the preservation of laryngeal function.

Based on a comprehensive analysis of various studies in the literature, it is evident that despite the early diagnosis of recurrences, about 30% of cases present with advanced lesions in the examined specimens [11]. This finding underscores a prevalent issue of understaging in recurrent cases, which can be attributed to both the biological characteristics of radiorecurrent tumors (chronic edema and fibrosis can obscure the real extent of the disease, more aggressive growth patterns, higher likelihood of extralaryngeal spread and subglottic extension, greater frequency of undifferentiated tumors with intravascular and perineural invasion) and the anatomical and structural alterations following laser surgery [63, 64••, 65••].

OPHLs should be considered a viable treatment option for recurrent LC, covering a broad range of indications, including rT1 and rT2 lesions with limited endoscopic visibility or transcommissural extension, rT2 lesions with impaired vocal cord mobility, and rT3 lesions with limited involvement of the paraglottic or pre-epiglottic spaces, as well as recurrent LC affecting the thyroid cartilage without extralaryngeal spread [66].

Regarding oncologic outcomes, studies investigating the efficacy of OPHL following RT and TOLM report local control rates between 70 and 95% at 2 years [67–73], disease-free survival (DFS) rates between 70 and 90% at 3 years [67–70, 72, 74], and overall survival (OS) rates between 70 and 90% at 5 years [67–70, 75•]. Despite these positive results, some cases may require salvage total laryngectomy. The organ-sparing rate stands at 85.2%, with a high decannulation rate, indicating successful airway management. However, laryngeal stenosis presents as a notable complication. Functional results show that most patients achieve efficient swallowing, though some may need gastrostomy. Phonatory outcomes vary, with some patients experiencing significantly altered voice quality [76•].

To sum up, in selected cases of recurrent LC, OPHLs offer a balanced therapeutic option that addresses both oncological and functional outcomes, promoting a conservative yet effective treatment paradigm and encouraging broader adoption across medical centers.

## Discussion

The renewed interest in partial laryngeal surgery, now particularly focused on therapeutic management for intermediate and advanced T stages, is attributable to a deeper understanding of the disease’s natural history, the low rates of cervical metastases in glottic cancer, and well-known patterns of laryngeal cancer spreading. These factors improve the use of OPHLs which can yield excellent oncological outcomes with a single treatment when surgical radicality is confirmed by pathology. Recent advancements in patient and tumor selection strategies have evolved beyond TNM classifications, incorporating endoscopic, functional, and radiological data to enhance safety and efficacy for conservative treatments even in advanced stages. Surgical innovations, such as the introduction of extended partial surgeries like STPLs, which have expanded the options for tumors extending towards the subglottic region with high risk of extralaryngeal spread, have paved the way for a modular approach to resection. This has broadened the scope of cases suitable for these surgeries.

A better understanding of high-risk areas for recurrence, especially regional recurrence at the VI level, has enhanced locoregional control through precise and targeted surgical interventions and elective dissections.

The variability in functional outcomes following partial laryngeal surgery, particularly in terms of voice quality and respiratory function, underscores the complexity of postoperative recovery. This variability highlights the critical need for continuous research into rehabilitation strategies to improve these outcomes. Advanced surgical techniques and postoperative care aim to preserve as much function as possible, but patient experiences can vary significantly. Corrective surgical procedures, especially injective laryngoplasties, have become crucial for refining functional results, particularly in voice quality and respiratory efficiency.

The controversies surrounding adjuvant therapies for advanced pT-stage tumors, with post-operative radiotherapy potentially negatively impacting functional results and without a clear oncological benefit in cases with positive margins, are significant [76]•.

## Conclusions

Considering published data and through a comprehensive analytical approach, it can be stated that open partial horizontal laryngectomies (OPHLs) currently occupy a crucial position in the conservative management of intermediate to advanced laryngeal cancer. They stand in competition with non-surgical organ preservation protocols regarding oncological outcomes and the preservation of laryngeal function. The selection of the most appropriate cases for OPHLs emerges as a critical factor. Today, surgeons possess more objective parameters to improve and comprehend this delicate aspect, which significantly relies on the physician’s experience.

The paramount objective remains the eradication of the tumor through a single treatment modality, thereby establishing OPHLs as a central intervention within the targeted therapy landscape.

The forced choice to resort to multimodal therapy due to unexpected upstaging should be considered a shortcoming of a single-modality surgical strategy aimed at preserving laryngeal function. This perspective highlights the significance of accurate preoperative assessment and the complexities involved in managing advanced cases, where the aim is to maintain organ functionality without compromising oncological integrity.

## Data Availability

No datasets were generated or analyzed during the current study.
